# Cryopreservation of *Echinococcus granulosus* Protoscoleces

**Published:** 2020-01

**Authors:** Alireza BADIRZADEH, Saber RAEGHI, Vahid FALLAH-OMRANI, Soheila ROUHANI

**Affiliations:** 1 Department of Parasitology and Mycology, School of Medicine, Iran University of Medical Sciences, Tehran, Iran; 2 Department of Laboratory Sciences, Maragheh University of Medical Sciences, Maragheh, Iran; 3 Cellular and Molecular Biology Research Center, Shahid Beheshti University of Medical Sciences, Tehran, Iran; 4 Department of Biotechnology, School of Medicine, Shahid Beheshti University of Medical Sciences, Tehran, Iran; 5 Department of Medical Parasitology and Mycology, School of Medicine, Shahid Beheshti University of Medical Sciences, Tehran, Iran

**Keywords:** *Echinococcus granulosus*, Cryopreservation, Dimethylsulfoxide (DMSO), Glycerol, Cystic hydatidosis, Iran

## Abstract

**Background:**

The main objective of the current study was to investigate on the cryopreservation of protoscoleces of *Echinococcus granulosus*, a causative agent of cystic hydatidosis in man.

**Methods:**

This study was conducted on isolated protoscoleces from hydatid cysts infected livers collected from slaughterhouse of Tehran, Iran in 2016. Viability of protoscoleces was evaluated by dye test. Cryopreservation of isolated protoscoleces in the presence of Dimethylsulfoxide (DMSO) and glycerol using a three-step cooling protocol involving an initial period at −20 °C, −80 °C and liquid nitrogen was performed.

**Results:**

The mean viability rate of 10% DMSO and 15% glycerol were 9% and 8% respectively. The protoscoleces of *Echinococcus granulosus* have been successfully thawed and recovered after 6 months storage in liquid nitrogen.

**Conclusion:**

Cryopreservation method needs to be improved for each species of helminthes and can be useful for other immunological and laboratorial studies.

## Introduction

Cystic hydatidosis is a worldwide parasitic disease, caused by the larval form of *Echinococcus granulosus*, highly prevalent in Middle Eastern developing countries such as Iran (1, 2). Ingesting the eggs of this parasite can infect humans and herbivorous animals, e.g. sheep and cow (3). Finding an appropriate method for long-time maintenance of this parasite in laboratory is very significant for research purposes (4).

There are many methods for detecting and preserving parasites in laboratory, which involve either keeping the larva or the adult form of the parasite (5–7). In the case of *E. granulosus*, working with adult worms is so risky, since its viable eggs are very infective and can easily infect the laboratory researchers. Therefore, in order to create safe research conditions, the cultivation of the organism needs to be strictly controlled (8). Storing the parasites at freezing state in laboratory is one of the useful methods to achieve this purpose (9).

Cryopreservation has a lot of practicable usage in molecular medicine, biology, microbiology and parasitology, with a massive capability for the storage of biological cell and tissue banking (10). Freezing parasites in liquid nitrogen is a well-established and extensively used technique by the Parasitologists for preserving parasites in a low-cost way, without any biological and morphological alterations or loss of functional features (9, 11). Another method for maintaining protoscoleces of *Echinococcus* spp. in laboratories is a serial passage between laboratory animals such as rodent. The problem with this method, is that firstly, the animals may suffer from the infection with protoscoleces of *E. granulosus* and secondly, the preservation of the animals requires extensive amount of laboratory space (4).

Cryopreservation technique has proven to be a successful method for the long-term storage of many protozoa and helminthes (10–11). However, there have been a very few studies on the application of cryopreservation method for *Echinococcus* spp. (12).

In this study, for the first time in Iran, we tried to examine an effective conventional slow cryopreservation method to construct a helminthes bank for *E. granulosus* protoscoleces, by using cryoprotective agent (CPA) of DMSO and glycerol in the liquid nitrogen.

## Materials and Methods

**Fig. 1:**
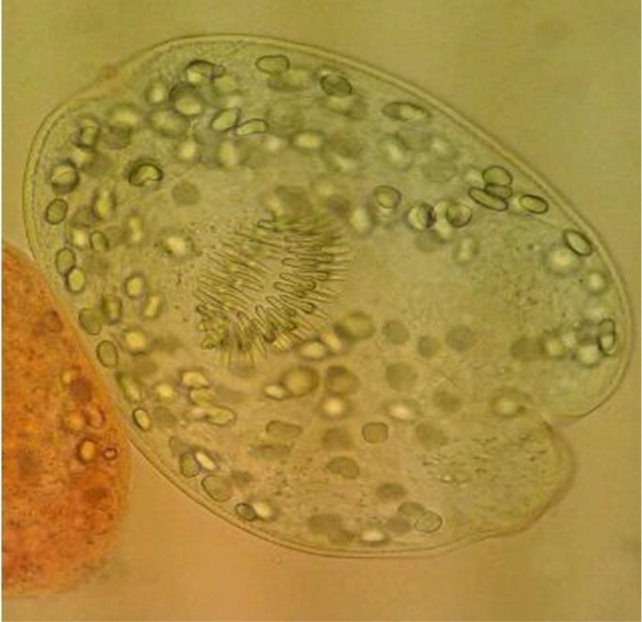
Live protoscoleces of *E. granulosus* which appeared to clear after thawing

**Fig. 2:**
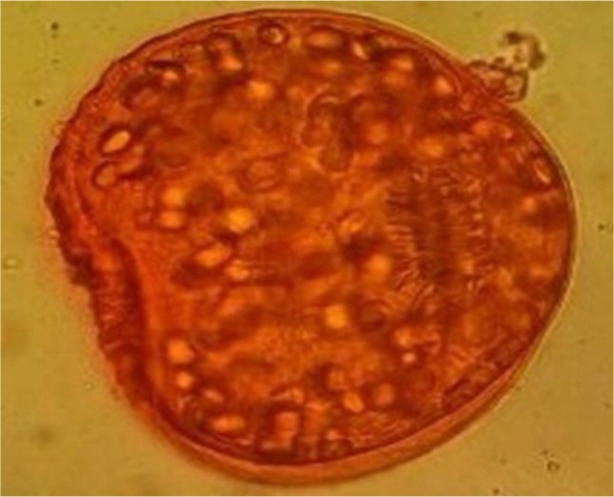
Dead protoscoleces of *E. granulosus* which appeared to red after thawing

This study was conducted in 2016 (Apr–Aug) in the Department of Parasitology at Shahid Beheshti University of Medical Sciences (SBMU), Tehran, Iran. Hydatid cyst – infected livers collected from the Maysam slaughterhouse of Tehran and transferred to the parasitology laboratory. The protoscoleces aspirated from liver in sterile condition by using a 10 ml syringe with a 22 gauge needle. After three times of washing with Hank’s solution (HBSS 40 ml), the viability of protoscoleces was evaluated by eosin 0.1% dye test. The live protoscoleces did not absorb eosin and appeared clear while the dead one appeared to be red (Figs. 1, 2). Dead and alive protoscoleces were counted by light microscope (Carl Zeizz Axiolab 50X, 100X). All pellets of protoscoleces form different hydatid cysts were mixed and then collected by quick micro centrifuge. After soft and gentle shaking of sediments, about 0.2 ml aliquots of protoscoleces were transferred into round-bottom containing of 0.8 ml of RPMI 1640 with screw-cap cryotubes (NUNC-Intermed, DK-Roskilde).

Dimethylsulfoxide (DMSO) and glycerol were used separately as cryoprotectants in our experimental procedures. In order to prepare different concentration of each cryoprotectant, the appropriate amount of each cryoprotectant was added to pellet of protoscoleces in RPMI 1640 where the final concentrations were reached to 5%, 10%, 15%, and 20 %.

At the beginning, the aliquots were kept for 15 min at 22–24 °C (room temperature) to allow completing glycerol diffusion whilst cryopreservation with DMSO should have been done immediately, in order to avoid the toxic and deadly effects of this cryoprotectant. The samples with both cryoprotectants were kept at 4 °C for 20 min before freezing in advance. Then, the cooling/freezing process was conducted on the basis of a three-step validated protocol; firstly, cooling for 30 min at −20 °C, afterward 30 min at −80 °C. Finally, they were directly soaked in liquid nitrogen at −196 °C for six months using a Chronos 80 storage system (Messer, D-Griesheim). The thawing and recovering of protoscoleces were precisely done on first, third and sixth month by gentle shaking of the cryopreserved samples in a water bath at 37 °C for 2–3 min until the ice of each cryotube was evaporated. This procedure gives a thawing rate of approximately 600–800 °C/min. The prepared samples were instantly diluted and washed in Hank’s solution at 37 °C for the purpose of getting rid of cryoprotectant substance. The pellet was precipitated by quick microcentrifuge and Hank’s solution was removed. Finally, RPMI 1640 was added to pellet as a nourishing medium of protoscoleces. Then, viability assay was evaluated by eosin 0.1% dye test as described above. One hundred protoscoleces were counted two times in two different cryotubes containing the aliquot.

## Results

The primary viability of protoscoleces was 99% before cryopreservation procedure. Table 1 shows the effect of type, concentration and time of cryoprotectants (DMSO and glycerol) on survival rate of *E. granulosus* protoscoleces. In this study, the best concentration of DMSO and glycerol for freezing of protoscoleces were 10% and 15% respectively. The concentration of 5%, 15% and 20% DMSO and 5%, 10% and 20% glycerol was not successful for cryopreservation protoscoleces. They had no motility and most of them were damaged (Fig. 2).

**Table 1:** Effect of type, concentration and time of cryoprotectants on survival rate of protoscolices of E. granulosus

***Cryoprotectants***	***Cryoprotectants concentration (%)***	***Survival rate (%)***			
		1^st^ Month	3^rd^ Month	6^th^ Month	Mean
DMSO	5	2	2	4	2.7
	10	10	8	9	9
	15	1	2	1	1.3
	20	1	0	0	0.33
Glycerol	5	1	2	2	1.7
	10	2	3	1	2
	15	9	7	8	8
	20	1	1	0	0.7

The mean survival rates of cryopreserved protoscoleces were 9% with 10% DMSO at −196 °C in liquid nitrogen and 8% with 15% glycerol (Fig. 1).

## Discussion

Today, using conventional techniques for the storage of parasites in laboratory animals or in vitro condition have led to some constraints such as, difficulty in isolation, loss of parasites, contamination with other opportunistic microorganisms and variation in bio-physiological features (13). Indeed, finding an alternative method like cryopreservation can profoundly decrease such restrictions (12).

Deeply freezing method was used by many researchers to preserve protozoan parasites in a viable state in liquid nitrogen (4, 12). Two methods have been used for cryopreservation of parasites; rapid and slow cooling. Protozoan parasites such as *Trypanosome cruzi* were successfully cryopreserved by rapid freezing method (12, 14). In this method, a cryotube containing the parasite is directly immersed into liquid nitrogen; another method for cryopreservation is slow freezing (4). *Entamoeba histolytica*, *Giardia lamblia* and *Trichomonas vaginalis* were cryopreserved by slow freezing (4, 15, 16). For controlling freezing rate (1–20 °C/min) in the slow method, programming freezer or alcohol bath can be used. Since 1980, only some experimental procedures were done on the cryopreservation of helminths, but no experiment in this area on *E. granulosus* (17).

To our knowledge, there are a few published data on cryopreservation used for cestodes (13). The cysticerci of *Taenia crassiceps* had been frozen at −55 °C with methanol (20%) as CPA, and then such cysticerci was able to proliferate in mice (18).

Cryopreservation in liquid nitrogen of larval *E. multilocularis* is possible under different experimental conditions. Final viability test was assessed after transferring the cryopreserved materials into the peritoneal cavity of Merinos (9). Metacestodes of *E. multilocularis* can be successfully stored by cryopreservation without failing their proliferative capacity in Meriones rodents as an intermediate host. The best proliferation rate in Merinos occurred when 10% glycerol was used as a cryoprotectant and a three-step freezing schedule was used (30 min at −28 °C, 30 min at −80 °C and transferred to liquid nitrogen) (9). In another study, a rapid method was used for the cryopreservation of *E. multilocularis* in liquid nitrogen wherein the metacestodes were remained infective for rodents after final process of cryopreservation (13).

In the present study, we examined two cryoprotectants in different concentrations. The best concentrations of DMSO and glycerol were 10% and 15% respectively. The freezing schedule was the same as the study mentioned above except the first step of procedure (30 min at −20°C) (9).

## Conclusion

Our study was done for the first time in Iran. In effect, since this was our first experience, it needs further trials and experiments. Furthermore, cryopreservation method needs to be performed and improved for each species of helminthes.

## Ethical considerations

Ethical issues (Including plagiarism, informed consent, misconduct, data fabrication and/or falsification, double publication and/or submission, redundancy, etc.) have been completely observed by the authors.
